# E-learning in medical education in resource constrained low- and middle-income countries

**DOI:** 10.1186/1478-4491-11-4

**Published:** 2013-02-04

**Authors:** Seble Frehywot, Yianna Vovides, Zohray Talib, Nadia Mikhail, Heather Ross, Hannah Wohltjen, Selam Bedada, Kristine Korhumel, Abdel Karim Koumare, James Scott

**Affiliations:** 1 The George Washington University, 2121 K St NW, Washington, DC, 20037, USA; 2 Georgetown University, 3700 O St NW, Washington, DC, 20057, USA; 3 Université de Bamako - Colline de Badalabougou, BP E2528, Bamako, Mali, West Africa

**Keywords:** E-learning, Low- and middle-income countries, Medical education, Resource constrained

## Abstract

**Background:**

In the face of severe faculty shortages in resource-constrained countries, medical schools look to e-learning for improved access to medical education. This paper summarizes the literature on e-learning in low- and middle-income countries (LMIC), and presents the spectrum of tools and strategies used.

**Methods:**

Researchers reviewed literature using terms related to e-learning and pre-service education of health professionals in LMIC. Search terms were connected using the Boolean Operators “AND” and “OR” to capture all relevant article suggestions. Using standard decision criteria, reviewers narrowed the article suggestions to a final 124 relevant articles.

**Results:**

Of the relevant articles found, most referred to e-learning in Brazil (14 articles), India (14), Egypt (10) and South Africa (10). While e-learning has been used by a variety of health workers in LMICs, the majority (58%) reported on physician training, while 24% focused on nursing, pharmacy and dentistry training. Although reasons for investing in e-learning varied, expanded access to education was at the core of e-learning implementation which included providing supplementary tools to support faculty in their teaching, expanding the pool of faculty by connecting to partner and/or community teaching sites, and sharing of digital resources for use by students. E-learning in medical education takes many forms. Blended learning approaches were the most common methodology presented (49 articles) of which computer-assisted learning (CAL) comprised the majority (45 articles). Other approaches included simulations and the use of multimedia software (20 articles), web-based learning (14 articles), and eTutor/eMentor programs (3 articles). Of the 69 articles that evaluated the effectiveness of e-learning tools, 35 studies compared outcomes between e-learning and other approaches, while 34 studies qualitatively analyzed student and faculty attitudes toward e-learning modalities.

**Conclusions:**

E-learning in medical education is a means to an end, rather than the end in itself. Utilizing e-learning can result in greater educational opportunities for students while simultaneously enhancing faculty effectiveness and efficiency. However, this potential of e-learning assumes a certain level of institutional readiness in human and infrastructural resources that is not always present in LMICs. Institutional readiness for e-learning adoption ensures the alignment of new tools to the educational and economic context.

## Background

In 2006, the World Health Organization (WHO) announced that fifty-seven countries lack 4.2 million health care workers, including medical doctors, nurses and allied health care workers [1]. In many of these countries there is a concerted effort to increase the health care workforce and its equitable distribution in underserved areas. As part of this effort, many of these countries are increasing student intake at existing medical schools, as well as building additional medical schools in rural areas. A major challenge to training increasing numbers of medical students, while preserving the quality of their training, is the shortages of faculty in most medical schools. In many low- and middle-income countries (LMIC) there exists a shortage of faculty, exacerbated by the need to increase medical doctor graduation rates [2]. In many instances, large well-established medical schools are expected to provide or share faculty with newly created medical schools. In such cases, e-learning provides an opportunity to extend faculty availability to new medical schools and reach distant students [3-5].

In this paper, we define e-learning to include all forms of electronically-mediated teaching. In other words, it is teaching and learning that is facilitated via information and communications technology (ICT), both inside and outside the classroom. Although e-learning tools have been used in many settings for a long time [6], evidence of their use in the pre-service education of medical professionals is limited, especially in LMICs.

This paper catalogues the ways in which electronically-mediated teaching methods have been utilized by medical schools in LMICs. It describes the challenges faced in implementing such programs and suggests avenues for evaluating their effectiveness in diverse settings, highlighting successful examples throughout. It also examines the effectiveness of these methodologies as reported in the original articles and the benefits to students and faculty from incorporating e-learning into a medical educational curriculum. Finally, it proposes a framework for the implementation of e-learning in resource-constrained settings.

### Definitions of e-learning terms

We begin by providing a list of definitions of the varied e-learning approaches. These are described in the Table 1. For each approach, we highlight implementation considerations for sustainable e-learning programs in relation to institutional-level investments. Note that what is provided in the table is, to a large degree, generalized to address needs in resource-constrained settings. However, we recognize that there is variability within such settings and the considerations described should not be thought of as prescriptive.

**Table 1:** Definition of e-learning terms and implementation considerations for resource-constrained setting

**Term**	**Definition**	**Faculty and student support services needed**
**Blended learning**	A mixing of different learning environments and approaches that often includes both face-to-face classroom methods and computer-mediated activities in and/or outside the classroom.	♦→ Curriculum design or re-design (at a minimum course-level) expertise
		♦→ Reliable and supported instructional technology tools available
		○ Learning Management System
		♦ Facilities for e-learning product development
		○→ Instructional technology tools training options
		○→ Tool availability for faculty and student use
**Pure e-learning / Fully-online**	Complete reliance on e-learning materials for use without any face-to-face classroom methods. The nuanced difference between pure e-learning and fully-online tends to refer to the delivery platform. Fully online implies reliance on a web-based solution while pure e-learning is independent of the delivery platform.	♦→ Curriculum design or re-design (at a minimum course-level) expertise
***(Terms tend to be used interchangeably)***		♦ Reliable and supported instructional technology tools available
		○ Learning Management System (at a minimum)
		♦→ Specialized tools based on subject matter availability and support
		○→ Instructional technology tools training options
		♦→ Facilities for e-learning product development
		○→ Professional staff available for production of e-learning solutions
		○→ Tool availability for faculty and student use
		♦→ Digital library resources
**Computer-assisted instruction / Computer-aided instruction / Computer-based learning / Computer-based training**	The use of instructional material presented by means of a computer or computer system to enhance instruction and facilitate interactive learning.	♦→ Commercial products available
***(Terms tend to be used interchangeably)***		
		○→ Budget for purchasing and support
		○→ Instructional technology tools training options
		○→ Licensing/Membership costs covered
		♦→ Facilities for e-learning product development
		○→ Professional staff available for production of e-learning solutions
		○→ Tool availability for faculty and student use
**Digital library**	An organized collection of electronic resources, including publications, webcasts, electronic books, etc., that can be accessed via computers on a Local Area Network (LAN) or a Wide Area Network (WAN).	♦→ Commercial products available
		○→ Budget for purchasing and support
		○→ Instructional technology tools training options
		○→ Licensing/Membership costs covered
		♦→ Reliable and supported digital library tools available
**Distance education / Distance learning / Distributed learning**	A field of education that focuses on teaching methods and technology for students who are not physically present in a traditional educational setting such as a classroom. Blended learning and pure e-learning can be thought of as examples of distance education. Distributed learning, although it tends to be used interchangeably, implies a more learner-centered approach to the design of instruction.	♦→ Curriculum design or re-design expertise
		♦→ Reliable and supported instructional technology tools available
		○→ Learning Management System (at a minimum)
***(Terms tend to be used interchangeably*)**		♦ Specialized tools based on subject matter availability and support
		○→ Instructional technology tools training options
		♦ Facilities for e-learning product development
		○→ Professional staff available for production of e-learning solutions
		○→ Tool availability for faculty and student use
		♦→ Digital library resources
**E-teaching**	Involves the use of electronic instructional materials in both face-to-face and virtual classroom situations, and often nurtures interaction with information, materials, and ideas.	♦→ Commercial products available
		○→ Budget for purchasing and support
		○→ Instructional technology tools training options
		○→ Licensing/Membership costs covered
		♦→ Facilities for e-learning product development
		○→ Instructional technology tools training options
		○→ Tool availability for faculty and student use
		♦ Reliable and supported instructional technology tools available
**Internet-based learning / Web-based learning / Online learning / Virtual education**	An educational approach that involves the use of the internet for delivering learning materials, and supports teaching and learning using various online resources.These terms tend to be used interchangeably among educators because of their reliance to an internet connection; however, they do refer to different technology approaches. For example, web-based learning relies on an internet connection and the use of a web-browser with appropriate plug-ins to run different applications while Internet-based applications require an internet connection but not a web-browser. The core differences among these terms are how they are implemented from an Information Technology/Systems perspective.	♦→ Reliable and supported instructional technology tools available
		○→ Training options available
		♦→ Reliable and supported networked infrastructure available
***(Terms tend to be used interchangeably)***		
**Learning Management System / Course Management System**	A web-based application for the administration, documentation, tracking, and reporting of educational programs. Other tools, such as digital libraries can be integrated as part of an LMS to make it a more robust learning environment.	♦→ Training options available
		○→ LMS how-to training
		○→ Integration of LMS in teaching and learning training
		♦→ Reliable and supported instructional technology tools available
***(Terms tend to be used interchangeably)***		
**Mediated learning**	More commonly known as “computer-mediated learning” and/or “ICT-mediated learning” involves the use of a computer that enables or facilitates teacher-student interactions. There is a subtle difference between computer-aided instruction and mediated learning around the structured nature of the interaction. Computer-aided instruction is produced by adhering to the instructional design process and tends to be implemented as part of formal or non-formal education while mediated learning allows for informal learning opportunities.	♦→ Reliable and supported instructional technology tools available
		○→ Training options available
**Mobile learning**	An approach that involves the use of mobile technologies so that learners can access instructional materials remotely for just-in-time learning. This often entails the use of smart phones or tablets.	♦→ Reliable and supported instructional technology tools available
		○→ Training options available
		♦→ Integration of classroom facilities with mobile learning solution options
		○→ Lecture-capture options
**Polling / Audience response system / Clickers / Keypads/ Student Response System**	This approach allows large groups of people to vote on a topic or answer questions using remotes, keypads and/or other mobile devices. The results are instantly made available to participants.	♦→ Reliable and supported instructional technology tools available
***(Terms tend to be used interchangeably)***		
		○→ Training options available
		♦→ Commercial products available
		○→ Budget for purchasing and support
**Simulation-based learning**	An approach to learning that mimic real systems and/or situations to learn through exploration, performing experiments, solving problems before implementing in the real world. E-learning simulations use information and communications technologies to create these environments so that learners can engage in individual and/or collaborative problem solving.	♦→ Facilities available for use by faculty and students
		♦→ Reliable and supported instructional technology tools available
		○→ Commercial products available
		▪→ Budget for purchasing and support
		▪→Instructional technology tools training options
		▪→Licensing/Membership costs covered
		○→ Custom-built products based on specific instructional needs
		▪→Staffing expertise for implementation, maintenance, and support
**Video Teleconferencing (VTC)**	A way to engage people at different locations in synchronous interaction. VTC includes video and audio feeds streaming in real time. Virtual classrooms can be conducted using VTC tools that allow for live teacher instruction and feedback via audio/video interactions, whiteboard sharing, polling, breakout sessions, etc.	♦→ Facilities available for use by faculty and students
		♦→ Reliable and supported instructional technology tools available
		○→ Commercial products available
		▪→Budget for purchasing and support
		▪→Instructional technology tools training options
		▪→Licensing/Membership costs covered
		○→ Open-source products available
		▪→Staffing expertise for implementation and support

♦ represents main item that is needed for faculty and student support services;○ represents the sub-item that is needed under the main item (♦);▪ represents the items needed under the sub-item (○).

A modified systematic literature review was conducted. To locate peer-reviewed documents, Academic Search Complete and PubMed databases were explored using specific search terms. To locate literature and reports that were not peer-reviewed, the search engine google.com was explored. Search terms were developed through preliminary research regarding types and uses of e-learning. Table 2 comprises search terms used, which were connected with the term “resource-constrained” and with the name of each LMIC as defined by the World Bank^a^. The list of LMIC we have used comes as per the classification of the World Bank and is based on finance of the country. Searchers used the Boolean operators “AND” and “OR” to restrict the search to potential articles of interest for this paper, and cover all possible combinations.

**Table 2:** Terms for e-learning and pre-service education of medical professionals

**E-learning terms**	**Pre-service education of medical professionals**
"blended learning"	"Residency"
"simulation-based learning"	"School of Medicine"
"computer-assisted instruction"	"Medical School"
"computer-based learning"	"Clinical"
"computer-based training"	"Medical Education"
"audience response system"	"Doctors"
"digital library"	"Meded"
"distance education"	"Medical Curriculum"
"distance learning"	"Postgraduate Medical Education"
"distributed learning"	"Post-graduate Medical Education"
"computer-aided instruction"	"Pediatric"
"Multimedia"	"Surgery"
"internet-based learning"	"Obstetric"
"medical e-learning"	"Basic Sciences"
"online learning"	"Student"
E-teaching	
"virtual classroom"	
"virtual education"	
"web-based learning"	
"E-learning"	
"learning management system"	
"course management system"	
"mediated learning"	
"mobile learning"	
"VTC"	
"video-based"	

This comprehensive search returned 803 peer reviewed articles and 51 grey literature resources for a total of 854 articles. Reviewers narrowed the collected list of articles by first evaluating their abstracts, keeping those which mentioned the use of e-learning to facilitate pre-service health education (including medical, pharmaceutical, nursing and dental) in any of the listed resource-constrained countries. Abstracts not specifically pertaining to one or more LMIC and the use of e-learning for pre-service health education were discarded. When an abstract’s relevance was not immediately apparent, reviewers read the full article in order to determine whether they should be kept. If any ambiguity remained, the article was evaluated by a committee comprising four reviewers who made a final decision.

All articles finally included in this study mentioned a specific e-learning intervention, or a system or idea meant to improve, implement or alter such an intervention, for pre-service medical, nursing, pharmacy or dental education, in a country listed by the World Bank as being LMIC; 124 peer reviewed articles and 14 grey literature resources fit these requirements. Additionally, bibliographies of all documents retained (peer-reviewed and grey literature) were reviewed as part of a “snowballing” technique to find further relevant resources, including other documents and relevant websites. In all, 138 documents were included in the paper.

### Findings

#### Types of articles on e-learning

**Figure 1:**
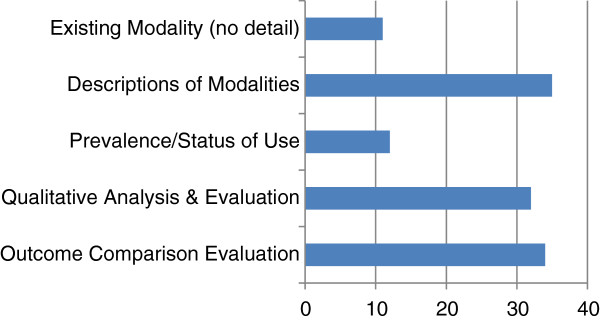
Types of articles on e-learning.

There were five types of articles found: i) articles that articulated the existence of an e-learning program or tool; ii) articles that described experiences in planning, implementing and managing e-learning which often included successes, challenges and lessons learned; iii) studies that assessed usage prevalence of certain modalities and e-learning tools; iv) studies that performed qualitative analyses designed to assess student and faculty attitudes towards e-learning modalities and; v) studies that focused on outcome comparisons in order to evaluate a modality or curriculum format (Figure 1).

The majority of articles described case studies and experiences with e-learning, highlighting methods for e-learning implementation in resource-constrained settings, its challenges and methods for sustainability. The second major set of articles evaluated the effectiveness of e-learning tools and compared the outcomes (often student scores) of such tools to the traditional in-classroom methods of teaching and learning (34 articles). Thirty-two articles presented qualitative analyses, while 12 articles used surveys to determine usage rates of e-learning modalities among faculty and students.

#### Countries

**Figure 2:**
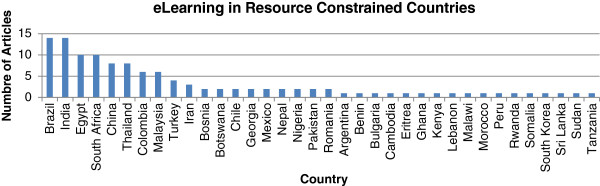
**Articles published about e-learning in pre-service health education, by country in which program studied took place.** Note that some articles refer to more than one country.

Of all the LMIC countries, Brazil, India, Egypt and South Africa are the countries that have published the most on e-learning in medical education. Of the 124 relevant articles found, 14 dealt with e-learning in Brazil, 14 in India, 10 in Egypt, and 10 in South Africa. While none of the articles located expressly assessed or mentioned the overall prevalence or usage of e-learning tools on an aggregate, country-wide level, the reviewed literature described many individual experiences from institutions in resource-constrained countries (Figure 2).

#### Health workforce

**Figure 3:**
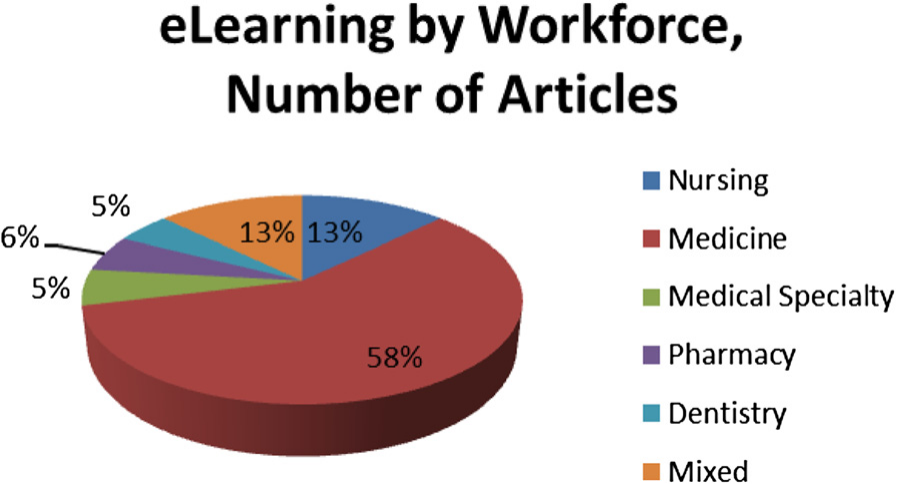
Peer-reviewed articles by faction of the human resources for health workforce.

While e-learning has been used by a variety of health workers in LMICs, the majority (63%) of the literature found focuses on physician training, of which only a small portion (5%) focused on specific post-graduate medical education including pathology, surgery, internal medicine, radiology, etc. Figure 3 illustrates the proportion of relevant articles by type of health profession.

#### E-learning strategies in resource constrained countries

A great variety of e-learning programs, tools and modalities used in medical, dental, nursing and pharmacy education was identified in LMICs. Thirty-six of the reviewed articles were descriptive articles lending insight into the design, implementation and lessons learned. Some examples included uses of ICTs as tools for accessing outside information, networked “telemedicine” systems allowing students to interact with educators at a distance, facilitators for problem- or case-based learning sessions, and blended teaching platforms, either in addition to or as replacements for face-to-face interaction between teachers and students.

**Figure 4:**
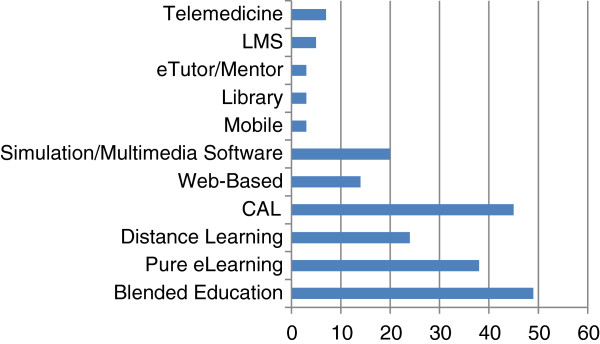
A cross-sectional view of e-learning modalities in the literature regarding health education in resource constrained countries.

Figure 4 aggregates the number of e-learning approaches that are described in the published literature. Blended educational approaches were most common; 49 articles presented various formal blended learning approaches. Computer assisted learning (CAL), specifically, comprised the majority of the blended learning approaches (45 articles). Three articles presented e-resources such as the eGranary Digital Library. Of the relevant 38 pure e-learning articles found (that is, e-learning used not in conjunction with other traditional techniques), the most commonly highlighted were simulations and the use of multimedia software (20 articles), web-based learning (14 articles) and eTutor/eMentor programs (3 articles).

#### Using e-learning due to faculty shortage

The articles that discussed using e-learning (Table 3) to address faculty shortages spoke of using e-learning to supplement faculty instruction at institutions [3,7-11]. The strategies used to do this included developing learning management systems through platforms such as Moodle, including interactive course materials and quizzes on specific topics. These materials included interactive text, videos, lectures, photos and animations. Virtual libraries were often utilized to house such materials. All of these activities were initiated in conjunction with traditional teaching strategies such as face-to-face lectures and labs.

**Table 3:** Using e-learning due to faculty shortages

**Country**	**Author**	**School**	**Educational topic**	**Reason**	**Type of e-learning**	**Challenges**	**Overall evaluation**
India	Agrawal [4]	Sanjay Gandhi Post Graduate Institute of Medical Sciences; Chattrapati Sahuji Maharaj Medical University	Clinical oncology, medical physics, radiobiology (all for radiation oncology training)	Expand reach	Videoconferencing sessions to connect understaffed radiotherapy departments	bandwidth (picture quality, time lag), presenter availability	Students found topics to be relevant, but remote trainees preferred in-person lectures due to technical difficulties with videoconferencing
	Kaliyadan [9]	Amrita Institute of Medical Sciences	Dermatology (structure and function of skin, morphology of skin lesions, psoriasis, leprosy, STIs)	Supplement	Digital self-learning modules with power-point presentations, videos demonstrating signs used in dermatological examination, interactive quizzes, crosswords and matching puzzles	Image and video quality	Students were liked the modules and were comfortable using them; there were no significant differences in knowledge acquisition from modules *vs.* traditional educational methods
Ghana	Adanu [10]	University of Ghana; Kwame Nkrumah University of Science and Technology	Biology (polymerase chain reaction), surgery (abdominal hysterectomy)	Supplement	Modularized programs specific to each topic with interactive text, videos, lectures, photos, and animations (created by faculty)	Electronic media could not be played on some computers	Medical students were able to access the modules easily and found them to be helpful educationally
Malaysia	Seluakumaran [11]	University of Malaya	Physiology	Supplement	Integrated the Moodle e-learning platform into undergrad physiology course (site included audiovisual resources and quizzes)	Poor internet connection and download speed	Student marks improved with the use of Moodle. Students were generally satisfied with the e-learning tool
South Africa	Mars [3]	University of KwaZulu Natal	All medical disciplines, including nursing	Expand reach	Videoconferencing of seminars, grand rounds, journal clubs, and research meetings to reach students at peripheral hospitals	Bandwidth (available, but cost is prohibitive); visual quality of slides	No difference in knowledge acquisition between e-learning and traditional learners; most instructors rated it as a good teaching tool
				Supplement	Moodle learning management system	Access to computers for regular use	
	McLean [8]	University of Natal Medical School	Histology	Supplement	Digital textbook and interactive multimedia packages on the eye and integumentary system		The majority of students thought that while CAI should supplement traditional learning it should not completely replace it. The interactive packages were valued more than the digital textbook.
Sri Lanka	Rajapakse [7]	University of Colombo	All undergraduate courses	Supplement	Established a virtual learning center: Moodle learning management system with interactive modules and assessments; virtual library	Staffing and time to produce learning material	The majority of students thought the LMS was useful. Most students use the virtual learning center to access the learning modules and the internet.
Turkey	Oz [5]	Istanbul University and Harran University	Basic sciences	Expand reach	Synchronous classroom conferencing to connect the two universities – video streamed the instructor, content from the document camera, PP presentations, and students in both locations; "boardcasted" two whiteboards that the instructor would use to write on	Connectivity; faculty learning curve	Students were positive about the course. Midterm and final exam scores were similar for students on both ends of the conferencing system.

Three articles reviewed e-learning strategies implemented to expand faculty reach, particularly at understaffed peripheral sites [3-5]. The primary strategy involved synchronously transmitting lectures, seminars, grand rounds and journal clubs via video-teleconference (VTC). Often, these sessions were recorded so that they could be shared asynchronously with individuals who were unable to join the session.

#### Using technology to support learning outside the classroom

The most common use of ICTs in health professions education was to access information that supplements formal teaching. The obvious example of this, in fact, a practice so common it seems authors neglect to publish on the topic, was when a student used an internet search engine to find articles, images, or slides regarding a given illness or medical practice. Kalita *et al.* studied the extent to which students at their institution in India utilized computer-based literature searches to supplement teaching of evidence-based medicine [12]. In a survey of 194 undergraduate and post-graduate students 89% stated that they used computer-based literature searches at least once per month to either prepare presentations (90.2%), carry out research (65%), or to research patient-related problems (60.3%) [12]. Following-up on this trend, in Egypt a digital library was made available to nursing students, and Taie explored the role of these libraries on student awareness, student knowledge and attitudes toward them [13].

Other articles described efforts to train students on how to use electronic databases. For instance, a Medical Information Retrieval and Application course instructed Chinese undergraduate medical students about medical literature searches, introducing students to the use of Medical Subject Headings (“MeSH” terms), the Chinese Biomedical Database, the China National Knowledge Infrastructure database, PubMed, Internet search engines, the Cochrane Library, and medical review writing [14].

In terms of the types of online resources used, Blankstein broadly cataloged some of the systems used in surgical education. He noted that listservs (which facilitate emails to large groups) have been in use by surgeons since the 1990s. Surginet is positively perceived by users, as “filling a niche” for informal, free exchange of ideas [15]. He also calls attention to online libraries, which he called easy to implement and use, to share resources, lecture slides and materials. According to Blankstein, Health Inter Network Access to Research Initiative (HINARI) and WHO, provide free access to journals which particularly improves access to resources for resource-constrained institutions [15].

#### E-learning in rural areas

Despite the lack of access to computers and internet in rural areas, several articles described e-learning programs in these contexts. One study described a Christian Medical College (CMC) and Tufts University partnership that successfully enhanced clinical training in a rural area of India using mobile technology [16]. The partnership analyzed several factors noting a spin-off benefit that distance learning was a powerful tool to encourage in-country retention of doctors. To address unreliable access to internet, several studies propose using blended learning with limited technological demands (mobile or low-bandwidth) in order to be more adaptable [16-18]. For example, mobile e-learning applications have been developed to enhance clinical training and provide support for students in rural areas where resources are scarcest [16,19].

#### Collaborations in e-learning

Partnerships in e-learning were common, especially given that the institutions mentioned in peer reviewed research were predominantly academic organizations. Often, schools in resource-constrained countries partnered with schools in wealthier countries. Such partnerships faced two challenges: i) the instructional material needed to be adapted to address realities of health care in developing nations, and ii) educational effectiveness was difficult to determine when communication, even via email, was minimal [20]. Despite the challenges, long-distance partnerships can be beneficial. For instance, the CMC-Tufts University collaboration mentioned above has created an e-learning infrastructure and established mobile learning for health program in India [16]. CMC installed Tufts University’s “TUSK” software (an enterprise knowledge and curriculum management system), and trained faculty, staff and students to utilize it. The US-centric software was systematically modified by CMC to orient content toward India’s needs, as well as for mobile use.

The University of Iowa, USA, collaborated with a number of schools to implement the WiderNet Project, which includes eGranary Digital Library delivering digital documents in resource-constrained countries without relying on the internet. eGranary provides access to web pages, audio, video and multimedia software through an institution's local area network (LAN), thereby reducing the individual users’ need to be connected to the World Wide Web in areas where such connections are difficult [18]. Although more systematic evaluation of this system, especially as it relates to implementation, training and sustainability costs, is needed in LMICs, user feedback has been positive. A Network Administrator from the Jomo Kenyatta University, Kenya, noted “the eGranary Digital Library has helped our students and lecturers in accessing academic materials which were not easily accessible due to limited bandwidth” [18]. Another comment, “the eGranary Digital Library has been a great bridge in the digital divide for us in the University of Jos in Nigeria” [18].

Given the limited number of microscopes in medical and biological institutions in underdeveloped areas, and the expense of investing in video microscopy systems, Silva-Lopes developed a less-expensive, high-quality, multimedia histology database using a combination of computer, intranet or internet, microscopes, and television [21]. Likewise, Missen depicted how implementing a locally-hosted eGranary Digital Library represented a relatively inexpensive and practical answer to the bandwidth challenge in developing countries [18].

The rationale for e-learning use in Francophone African countries is very similar to that of Anglophone African countries. Educational institutions often partner with other specialists or organizations in France and Belgium, wherever appropriate, for development and implementation assistance.

#### Effectiveness

Articles that examined the effectiveness of e-learning tools fell into quantitative comparisons and qualitative analyses.

##### Quantitative comparison evaluations

**Figure 5:**
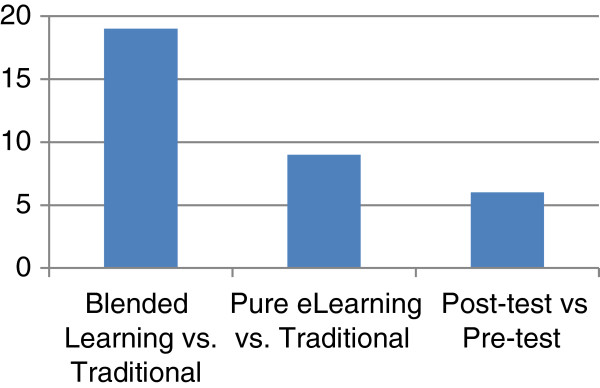
Outcome comparison approaches concerning the evaluation of e-learning methods.

The most common strategy to assess tools involved outcome comparison studies, whereby student test scores were compared between traditional and e-learning modalities (Figure 5). The majority of studies that compared blended e-learning methods to traditional approaches produced either promising results or no statistically significant differences in outcomes between the different modalities. They indicated that blended learning environments are effective for conveying medical knowledge and developing practical competencies. Comparisons involving pure e-learning approaches to traditional teaching also produced favorable results. Even studies that compared pre-test with post-test scores to evaluate the effectiveness of interventions demonstrated significant improvement in subject matter competencies.

Only one of the articles presented evidence against the effectiveness of e-learning technologies in the short-term. In this study students in a traditional methodology group performed better in the short-term compared to a group using computer-assisted instruction [22]. That said, the study found no statistically significant difference in longer-term retention of information three weeks after completion of the course.

##### Qualitative analysis evaluations

Another method of evaluating the effectiveness of programs involved the qualitative assessment of student and/or faculty attitudes towards the e-learning program. Utilizing survey questionnaires, studies revealed students reporting that the use of a blended mode of education, including computer-assisted learning with the use of the internet, multimedia, online lecture notes and quizzes, together with face to face lectures, practical projects and group work, was beneficial and promoted better quality of education. Students who participated in pure e-learning environments, such as emersion through simulation, multimedia programs or distance learning courses, also reported positive feedback and noted that these tools facilitated their understanding of curriculum material.

#### E-learning to improve health worker retention

Enthusiasm to implement e-learning tools is evident worldwide. Most studies report enthusiasm from distance learning students, as well as high demand for e-learning programs to address shortages of healthcare workers and improve retention rates. There was evidence in the literature that e-learning is a useful tool for overcoming barriers to access for health professions’ training. At least four studies reported a majority of students and faculty praising e-learning programs as effective and creative tools for developing regional capacity and for fulfilling the need for more healthcare workers in developing countries [20,23-25]. At least one study purported that blended learning methods of delivering education encouraged retention of current health workers by providing professional development that could not otherwise be attained [23]. The same study reported increased political will from the Tanzanian government to implement distance learning programs [23].

#### Cost-effectiveness

None of the studies evaluated the cost-effectiveness of e-learning tools. Also, cost-effectiveness assessment was not dealt with in-depth in the reviewed articles. Articles that mentioned specific costs for their respective interventions noted that these costs were not passed on to students with respect to the amount of use, because the schools absorbed that cost. In other words, several e-learning options, including simulations and learning management system course materials, provided students with unlimited access at no additional cost to them. One study mentioned web course tools (WebCT)^b^, a learning management system, as “probably the most convenient, efficient and least expensive means of delivering digitized resources to students [26].” Some studies of distance learning programs mentioned cost advantages in terms of time saved, most commonly as a result of the flexibility of these programs. In short, e-learning and other distance learning techniques can affect ‘economies of scale’, rather than outright cost savings. While the costs for production of e-learning modules, materials and infrastructure are substantial, such costs are reduced on a per-student basis when programs are provided to large and repeated classes of learners [27]. In the long-run, well-conceived programs present cost-effective mechanisms to disseminate more information to students. In Brazil, for example, Silva-Lopes noted that there were a limited number of microscopes in medical and biological institutions in underdeveloped areas, video-microscopy systems were expensive to implement, and there was limited space available for pictures in books. A less-expensive e-learning response established a high-quality histology database by creating a multimedia laboratory using a combination of computer, intranet or internet, microscopes, and television [21].

#### Challenges in implementing e-learning in medical education

Seven articles expressly identified inadequate infrastructure as a challenge when implementing e-learning in resource constrained countries [4,9,23,28-32]. Their major points include limitations in bandwidth, which often contributed to slow speed and low quality of videos or visual outputs [4,30-32], difficulties reading or watching content from a computer screen [9,29,30], slow speed of downloading from the internet, [20,28,31] inadequate computer facilities, limited access to computers [28], and frequent electrical power failures [28]. Of note, the challenge of bandwidth availability is rapidly being addressed in even the most resource-challenged areas. According to Brookings, bandwidth is expected to increase by 2,400% in East Africa while costs should be reduced by almost half at the completion of undersea cables currently under construction [33].

Lack of face-to-face interaction was also a challenge addressed in four articles [24,30,34-40]. This challenge was especially seen as prevalent among pure e-learning and distance learning approaches. The lack of face-to-face interaction contributed to professional isolation and decreased learning experiences [24], and made it difficult to engage participants in discussion [35]. However, efforts were clearly focused on improving existing programs, and on developing creative alternatives to meet the needs of students informed by various evaluations. eMentoring and eTutoring software, for example, have evolved to incorporate more interaction between students and facilitators for a more effective blended learning environment with valuable feedback mechanisms [31,41]. Mobile e-learning applications have been developed to provide support for students in rural areas [16,19].

Other challenges identified in the literature included: inadequate numbers of skilled personnel such as instructional designers or instructional technologists to support e-learning programs [28,42], working across time zones (when conducting real-time distance learning) [16,20], financial costs of implementing and maintaining e-learning infrastructure [24,32], and the time commitment required of teachers to commit to the experience [24,35,43]. One study that described the use of a virtual patient simulation system in Colombia mentioned “that the development and maintenance costs are crucial to the successful implementation and use of an application” [44].

Tailoring e-learning to meet country-specific realities, cultures and languages was a major challenge highlighted in the articles, five of which are notable [20,24,42,43,45,46]. Adapting educational material to e-learning is already difficult, but medical material must also be adapted to fit a country’s healthcare realities and to preserve idiomatic meaning in different languages. Educational materials must be culturally relevant and language appropriate: tailoring materials to reflect country-specific health care realities [20], gathering challenging, real-world cases despite often incomplete content [42,45], and uncertainty about language requirements or difficulty expressing thoughts in another language with the same message as in one’s own language [43].

## Discussion

The need for increasing the number of health care professionals in developing countries, and the difficulties in accomplishing this with limited faculty and institutional resources, has been well-documented [1,47]. E-learning models have attracted much attention in recent years as potential ways to meet the shortages as evidenced by the literature reported in this paper. Medical schools and other health education institutions, even those in the most resource limited areas, will be faced with the challenges of adopting appropriate e-learning solutions into the educational process. Over the next several years the question is not whether e-learning will be a component of health education, but rather what is e-learning best used for within institutions, when and how to implement e-learning successfully, and how we will ensure a beneficial effect on our learners and faculty.

E-learning solutions do not come in a “one size fits all” package that will work in all settings. In fact, the challenge when implementing e-learning is to ensure that its integration takes into consideration local context and accounts for specific instructional needs [20]. Before embarking on adoption of an e-learning solution, it is prudent for an institution to examine its readiness and needs.

In the findings section we described some of the challenges in implementing e-learning in medical education noting the substantial costs associated with the hiring of skilled personnel to provide instructional support, the production of e-learning materials and the infrastructure. To take advantage of possible economies of scale, an e-learning solution needs to be adopted strategically, for example for large and repeating classes. We propose that one way to move forward in addressing the challenges of implementing e-learning for medical education in resource-constrained settings is through a strategic examination of the elements that contribute toward e-learning solutions.

Before an institution formulates an e-learning strategy, it is important to examine and identify institutional capacity to ensure that what is being considered within an e-learning strategy is advisable, feasible and sustainable. Questions such as the following can be used to guide the process:

Where are we?

· Is the institution ready to develop, implement, and maintain an e-learning program?

· What is the institutional buy-in for such a program to succeed (what does the institution plan to supply to support programmatic success?

· What infrastructure to support e-learning exists now and how reliable are those resources?

Where are we going?

· What exactly are we trying to accomplish with the adoption of e-learning into our curriculum? Is this being used to provide new educational material or is it being used to increase the outreach to more students and/or students in remote sites?

· What are the goals of the e-learning program both short-term and long-term?

How do we know we have gotten there?

· What are the key indicators for success?

**Figure 6:**
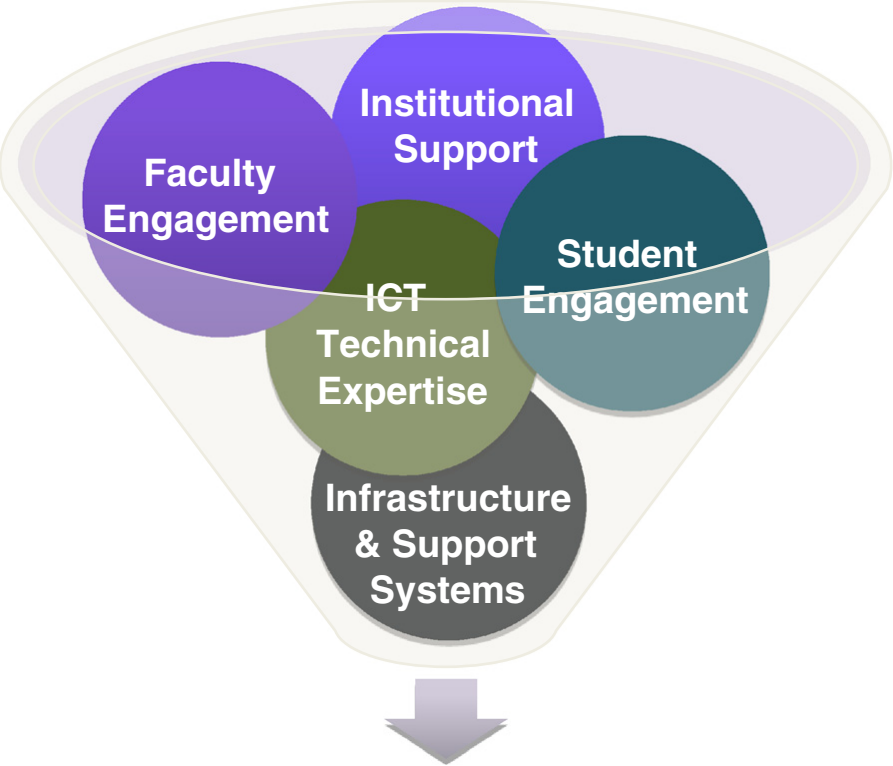
E-learning strategy.

With the above guiding questions in mind, we have identified from the literature the following elements of an e-learning strategy (Figure 6): *institutional support*; *faculty engagement*; *ICT technical expertise*; *faculty engagement*; *infrastructure and support systems*; and, *student engagement*. We describe each of the above elements more fully below.”’

### Institutional support

Institutional support is critical in sustaining an e-learning program independent of whether such a program is managed at the departmental or college level, or across an institution. Initial financial and human resources investments are substantial. To be successful, e-learning must be integrated into the curriculum, treated not simply as a delivery medium but as an environment that supports learning. Faculty must be supportive, feeling that the quality of teaching is not being compromised, but that e-learning is a way for students to gain access to instruction otherwise unavailable. Assessing institutional capacity for developing, deploying and sustaining an e-learning program is a necessary part of the process that focuses an e-learning strategy directly to meeting the needs of the institution [44]. It answers questions such as:

· What is the best use of e-learning, given our resources, for the enhancement of the education of our students?

· In the short term and in the long term, what effect will this have on faculty time and effort and how can we use this to deliver education more efficiently?

· What is available; what do we need developed; is this information available through e-learning or would it need to be developed; and are we able to develop these ourselves or do we need external support?

### ICT technical expertise and faculty engagement

A team approach to e-learning is important since there is often no single individual with the depth of expertise needed in all aspects of e-learning and of a health care curriculum to implement a program alone. Faculty must guide the development of the curriculum and courses which would be part of an e-learning program, working closely with ICT technical experts to create the mechanism [28,42]. In order to be able to build an e-learning program, ICT technical experts must understand the outcomes expected. This is especially true if the intent is to build e-learning environments. Faculties provide guidance and direction in terms of the curriculum and serve as spokespeople for the overall program. Thus, the roles of faculty and ICT experts should be identified and described before beginning [30]. As part of the process of identifying needs regarding an e-learning strategy, questions such as the following are answered:

· Is there an identified faculty champion for this project and what are the roles and responsibilities of a faculty lead?

· Are there incentives available for faculty to use e-learning? If not, should there be?

· Are there common incentives for faculty leads and IT leads to work together in creating an e-learning program?

· Will the adoption of new e-learning technologies, in a particular course or in the overall curriculum, require more, less or a similar amount of faculty time and energy compared to current educational delivery? If it requires more faculty resources, is the benefit of the e-learning additions worth the investment?

These types of questions help to identify existing capacity within an institution as well as gaps.

### Infrastructure and support systems

It is necessary to have a solid infrastructure that supports e-learning as a program [9,23,28,29]. Infrastructure requirements depend on the type of program that is envisioned. Infrastructure can be thought of from both the hardware needs, such as a network, computers and other equipment, and the software needs that include applications such as a learning management system, digital library, virtual lab and more. Infrastructure decisions should take into account both existing and future service needs. An institution must also account for the type and level of support that will be made available to those who use the services. If a learning management system is implemented, questions such as these should be answered:

· What kind of training should be in place for users?

· Will there be a Helpdesk for all users? What hours will it be available?

· How will the hardware be maintained? Is there a system administrator who will be responsible for upgrades, etc.?

· How much redundancy should there be to ensure a certain level of access and support?

Technical support is an essential part of building a successful e-learning strategy with long-term implications in terms of financial and human investments. Without support systems in place, an e-learning program may seem to succeed initially but most likely will not be sustainable.

### Faculty engagement and student engagement

To ensure that an e-learning strategy engages faculty and students appropriately, it is critical to determine how instructional development (the process of design and production of e-learning materials based on curricular needs) will be handled [8,28-30,36,37,39,44]. The established support system regarding teaching and learning at an institution plays an important role as the instructional development issues addressed are similar across institutions and contexts. These issues include: intellectual property and copyright issues; faculty incentives (financial, recognition); faculty development; and, student contributions and privacy.

It is equally important to assure that students are prepared for the introduction of e-learning into the curriculum. Do students have the technical skills needed to fully participate in e-learning and do they have reliable access to the technology needed? Will individuals recognize that e-learning material has the potential to greatly enhance their learning experience? Will they feel empowered to fully participate and are their cultural norms that must be accommodated to make sure that occurs?

Given the constraints faced in medical education within the context of LMICs and the cost associated with any e-learning implementation, it is prudent to account for a monitoring system as part of an e-learning strategy to capture, analyze and report the return on investment. Even though e-learning can be conducted independent of time and place, the associated costs of implementation cannot. Technology-enhanced learning solutions change at a much faster pace than established policy and institutional processes. This being the case, it is prudent for schools to consider carefully whether e-learning is going to be implemented for both the basic science as well as the clinical curriculum. In many LMICs, the clinical training takes place within state-run hospitals which raises serious considerations in terms of who bears the cost for supporting e-learning within clinical settings. In such a scenario, the implications of implementing an e-learning program for an entire medical education curriculum cut across sectors. Due to these cross-cutting implications, it is critical for those involved to establish mechanisms that engage decision-makers across institutional and possible ministerial boundaries.

As we have seen from the literature, e-learning and other distance learning modalities may offer learning opportunities where there is limited access to teaching in a specific field either because of a lack of qualified faculty or geographically distant faculty. The education world is increasingly interfacing with the IT world. Important lessons have been learnt regarding the most successful interactions between the two. Once each has learned from the other, can e-learning truly be deemed a realistic option for medical schools in pursuit of increasing the capacity and retention of health workers in resource-constrained settings?

## Conclusions

E-learning in medical education is a means to an end, rather than the end in itself. Our review demonstrates how e-learning is utilized and adapted in various LMIC countries to encompass a diverse range of competencies in basic science, medical knowledge, specialization and clinical practice. A great variety of methods ranging from digital libraries to more complex distance learning networks, multimedia software, learning management systems, virtual simulations, mobile applications and other e-resources, are being used. Studies suggest that e-learning may be effective for increasing capacity in rural settings. Although there is cautious optimism about e-learning, more experience will be forthcoming and more analysis is warranted especially around policy-related issues that surface through an institutional e-learning implementation. Policy-related issues include, but are not limited to, information and communications technologies such as network security, bandwidth and storage solutions. There is a further need for policies in relation to content and knowledge management of e-learning artifacts involving considerations around copyright and intellectual property as well as accessibility. Educators and institutions must identify appropriate e-learning tools for use in resource-constrained settings, analyze the effect of these modalities in decreasing the already constrained faculty time, understand the practicality and cost-effectiveness of e-learning use in resource constrained countries, and develop financial models for the sustainability of e-learning solutions. Only when the appropriateness, feasibility and true costs of e-learning tools and methodologies are understood in the context of LMICs, can their impact upon the health of country populations be realized.

## Endnotes

^a^For the purpose of this paper, “resource-constrained” will be defined economically, as referring to a country listed by the World Bank as having low- or middle-income. The relevant terms used include: “low income”, "resource constrained", Afghanistan, Albania, Algeria, Angola, Argentina, Armenia, Azerbaijan, Bangladesh, Belarus, Belize, Benin, Bolivia, Bosnia and Herzegovina, Botswana, Brazil, Bulgaria, Burkina Faso, Burundi, Cambodia, Cameroon, Central African Republic, Chad, Chile, China, Colombia, Congo Democratic Republic, Congo Republic, Costa Rica, Cote d'Ivoire, Cuba, Democratic Republic of Korea, Dominican Republic, Ecuador, Egypt, El Salvador, Eritrea, Ethiopia, Gabon, The Gambia, Georgia, Ghana, Guatemala, Guinea-Bisau, Haiti, Honduras, India, Indonesia, Iran, Jamaica, Jordan, Kazakhstan, Kenya, Kosovo, Kyrgyz Republic, Latvia, Lebanon, Lesotho, Liberia, Libya, Lithuania, Macedonia, Madagascar, Malawi, Malaysia, Mali, Mauritania, Mexico, Moldova, Mongolia, Morocco, Mozambique, Myanmar, Namibia, Nepal, Nicaragua, Niger, Nigeria, Pakistan, Panama , Paraguay, Peru, Philippines, Romania, Russian Federation, Rwanda, Senegal, Serbia, Sierra Leone, Somalia, South Africa, Sri Lanka, Swaziland, Sudan, Syrian Arab Republic, Tajikistan, Tanzania, Thailand, Togo, Tunisia, Turkey, Turkmenistan, Uganda, Ukraine, Uruguay, Uzbekistan, Venezuela, Vietnam, West Bank and Gaza, Yemen, Zambia, Zimbabwe.

^b^As of 2006, WebCT was acquired by Blackboard Inc. and has been phased into the Blackboard Learning System.

## Abbreviations

CAL: Computer assisted learning; CM: Christian Medical College; HINARI: Health Inter Network Access to Research Initiative; ICT: Information and communications technology; LAN: Local area network; LMIC: Low and middle income country; VTC: Video teleconference; WebCT: Web course tools; WHO: World Health Organization.

## Competing interests

The authors of this manuscript declare that they have no competing interests.

## Authors’ contributions

SF conceived the project idea, participated in its design and coordination, and drafted the manuscript. YV conceived the project idea, participated in its design and coordination, and drafted the manuscript. ZT helped to draft the manuscript. AKK, NM collected and analyzed literature review data and drafted the manuscript. HR collected and analyzed literature review data and drafted the manuscript. HW collected and analyzed literature review data and drafted the manuscript. SB collected and analyzed literature review data and drafted parts of the manuscript. KK collected and analyzed literature review data and developed manuscript figures and tables, and drafted related manuscript. JS reviewed and drafted manuscript. All authors read and approved the final manuscript.

## Authors’ information

Seble Frehywot and Yianna Vovides are First authors
